# Medical Versus Surgical/Endoscopic Management of Malignant Bowel Obstruction in Patients With End-Stage Gynecologic Cancer: A 12-Year Single-Center Experience

**DOI:** 10.7759/cureus.104202

**Published:** 2026-02-24

**Authors:** Haruho Kodama, Yoko Aoyagi, Kentaro Kai, Kensuke Fukuda, Yohei Kono, Yoshimi Goto, Tomonori Yamada, Eri Obata, Shizuka Iwabuchi, Eiji Kobayashi

**Affiliations:** 1 Department of Obstetrics and Gynecology, Oita University Faculty of Medicine, Yufu, JPN; 2 Department of Gastroenterology, Oita University Faculty of Medicine, Yufu, JPN; 3 Department of Gastroenterological and Pediatric Surgery, Oita University Faculty of Medicine, Yufu, JPN; 4 Hospital Information Center, Oita University Hospital, Yufu, JPN

**Keywords:** end-of-life, endoscopic management, gynecologic cancer, malignant bowel obstruction, medical management, surgical management

## Abstract

Introduction: Malignant bowel obstruction (MBO) is among the most challenging aspects of late-stage gynecologic cancer care. Surgical/endoscopic interventions carry high complication risks in patients with carcinomatous peritonitis and cachexia. This study examines the impact of surgical/endoscopic interventions on survival time in patients with gynecologic cancer.

Methods: We conducted a 12-year retrospective chart review (2013-2024) of all patients with MBO associated with cervical, endometrial, or ovarian cancer who received their initial treatment at our institutions. This MBO cohort was divided into two treatment groups: (i) medical management alone (nasogastric tube, long intestinal tube, octreotide) and (ii) surgical/endoscopic management (percutaneous endoscopic gastrostomy, metallic stent, bypass surgery, stoma formation). The primary outcome measure was 90-day survival from MBO onset; secondary outcome measures included the proportion of patients who, after MBO treatment, recovered oral intake and were discharged home. We compared survival times using Kaplan-Meier analyses and log-rank tests, and analyzed categorical data using the chi-square and Fisher's exact tests. Variables affecting survival time were assessed using the Cox proportional hazards model.

Results: A total of 45 cases of MBO were identified among 1,085 gynecologic cancer patients (4.1%). Of these, 95.5% (43/45) underwent some form of medical management. Of these 43 patients, 16 underwent surgical/endoscopic management. The proportion of patients who regained oral intake and were discharged home after MBO treatment was significantly higher in the surgical/endoscopic group (p < 0.001 for both outcomes). In multivariate analysis, surgical/endoscopic treatment was associated with more prolonged survival (hazard ratio (HR) 0.280; 95%CI 0.104-0.754, p = 0.012), whereas ascites severity was associated with shorter survival (HR 2.252; 95%CI 1.015-4.998, p = 0.046).

Conclusion: At the end-of-life care for patients with late-stage gynecologic cancer and MBO, we found that surgical/endoscopic intervention better enabled resumption of oral intake and discharge to home without affecting survival time.

## Introduction

Malignant bowel obstruction (MBO) is among the most challenging aspects of late-stage, incurable cancer care [1]. Because the patient’s life expectancy is uncertain, their goals and preferences must be considered when selecting a treatment [2,3]. The treatment for MBO associated with end-stage cancer care has been classified in various ways in the literature. Still, it is generally divided into two types: medical (non-surgical) and surgical [1,4]. Medical or non-surgical treatment includes pharmacologic agents (e.g., octreotide (OCT)) and decompression of the stomach (nasogastric tube (NGT)) and the small intestine (long intestinal tube (LT)) [5,6]. Surgical or invasive treatments include percutaneous endoscopic gastrostomy (PEG), self-expanding metallic stent insertion (MS), intestinal bypass surgery (BS), and stoma formation (SF) [7,8]. However, although medical treatment is noninvasive and can be effective to some extent, approximately half of patients fail to respond [9]. In contrast, some researchers have reported that surgical treatments are more promising than medical treatment [10]; however, their morbidity and mortality are not negligible [11]. Surgical treatment can cause life-threatening complications, such as perforation and sepsis, especially in critically ill patients [12,13]. Additionally, clinical practices vary widely across institutions, making it difficult for us to grasp the big picture. While there are society guidelines and recommendations on MBO, clear criteria for transitioning from medical treatment to surgical or invasive treatment, or for selecting a surgical candidate at the outset, are lacking [14,15].

Gynecologic MBOs are common and are encountered frequently by gynecologic oncologists in clinical practice [16]. The overall prevalence of MBO ranges from 3% to 15% among all gynecologic cancer patients, with the highest prevalence of up to 50% in patients with ovarian cancer [1]. By primary site, the ovary and the endometrium are the first (16-29%) and sixth (3-11%) most frequent sites in Western European countries [17]. However, the quality and quantity of these data are biased with regard to Western European countries. In Japan, two distinct sociodemographic factors should be considered. First, both the incidence and mortality rates of cervical cancer in Japan are the highest among the Group of Seven (G7) countries, due to a historically low anti-human papillomavirus (HPV) vaccination rate (< 1%) and cancer screening rates (35-40%) [18]. Second, the proportion of deaths at home has remained low, at about 14%, which is less than half that in Western European countries [19]. Nevertheless, comprehensive epidemiological research on MBO management in Japan is lacking.

The purpose of this study is to review our experience and assess the impact of medical, surgical, and endoscopic interventions on survival in patients with gynecologic MBO. We also explored the relationship between treatment type and the proportions of patients who recovered oral intake and were discharged home as end-of-life care outcomes.

## Materials and methods

Study design and setting

This was an observational, retrospective cohort study conducted at Oita University Hospital, Yufu City, Japan, the only university hospital in Oita Prefecture, which has a population of approximately 1.7 million. Oita University Hospital is a tertiary, educational, and referral hospital and does not have specialized palliative care wards. Palliative care was provided to patients by the attending physician and the palliative care consultant team, comprising an anesthesiologist, psychiatrist, nurse, pharmacologist, medical social worker, dietitian, and psychologist.

Study population

A 12-year retrospective chart review (2013-2024) was conducted of all patients newly diagnosed with cancer. We then identified the study cohort using the inclusion and exclusion criteria below.

Inclusion criteria were as follows: patients with epithelial cervical, endometrial, and ovarian cancer who received their initial treatment at our institution, patients with recurrence after or during front-line treatment, and patients receiving therapy for MBO via NGT, LT, OCT, PEG, self-expanding MS insertion, intestinal BS, and SF.

Exclusion criteria were as follows: patients without recurrences after front-line therapy (n = 800), patients without any MBO treatment defined above (n = 236), and patients with MBO who initially presented with pneumoperitoneum and required emergency SF (n = 4). The diagnosis of MBO was initially made by the attending physician based on the patient's clinical history and symptoms, and was subsequently confirmed by computed tomography (CT). The diagnosis was then confirmed by consultation with the gastrointestinal surgeon and gastroenterologist.

**Figure 1:**
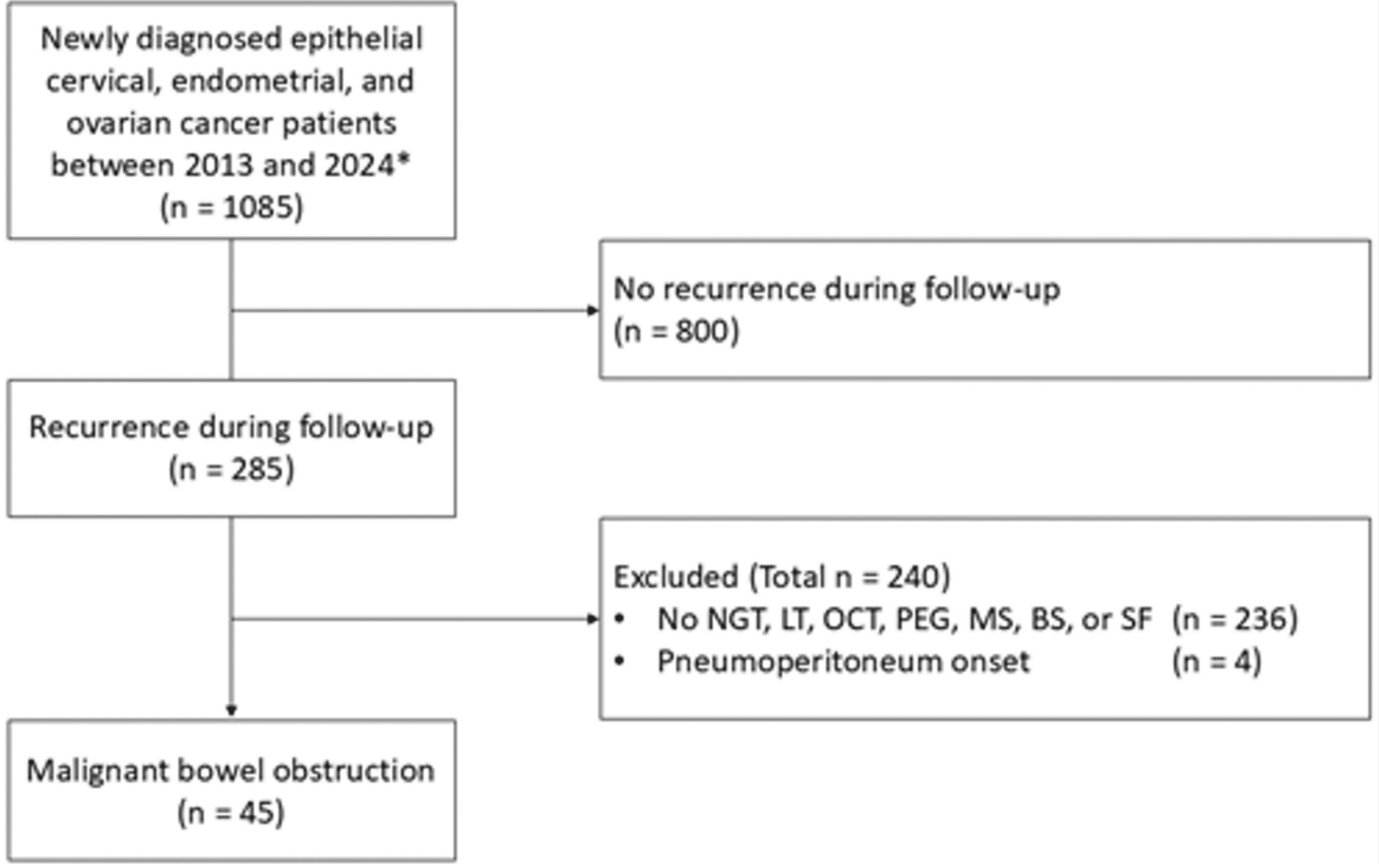
Flow diagram showing selection of study population *Ovarian cancer includes fallopian tube and primary peritoneal cancer. NGT, nasogastric tube; LT, long intestinal tube; OCT, octreotide; PEG, percutaneous endoscopic gastrostomy; BS, bypass surgery; SF, stoma formation

Finally, 45 of the 1,085 gynecologic cancer patients (4.1%) treated were identified as having MBO and thus included in the analysis. Figure 1 shows the process of selection of the study population.

Outcomes

The primary outcome measure was 90-day survival, defined as the interval from MBO onset to death from any cause. The data cutoff date for survival analysis was November 4, 2025. The survival for subjects alive at the last contact or lost to follow-up was calculated from the date of the previous contact. The secondary outcome measures in our study cohort were the proportions of patients who resumed oral intake and were discharged home.

Variables 

Performance status (PS) was defined according to the Eastern Cooperative Oncology Group (ECOG) Performance Status Scale [20]. The staging was established using the latest International Federation of Gynecology and Obstetrics (FIGO) classification system for the primary disease at diagnosis [21]. The obstruction site was initially categorized based on the radiologic report and subsequently reviewed and modified by two researchers (KF and YK). Medical treatment in this study was defined as any use of NGT, LT, or OCT. Surgical treatment was defined as the patients who underwent endoscopic procedures (PEG and MS) and/or surgical procedures (BS and SF)

Statistical analysis

We used Venny 2.1.0 to generate the analytical diagrams [22]. For univariate analysis, we compared time to death after MBO onset using Kaplan-Meier estimates and log-rank tests. Chi-square and Fisher’s exact test were used for categorical values. For multivariate analysis, we used the Cox proportional hazards model to assess the impact of all variables on overall survival (OS) after MBO onset. The results of the multivariate analysis are expressed as hazard ratios (HRs) with 95% confidence intervals (CIs). A p-value of < 0.05 was considered statistically significant, and all tests were two-sided. All statistical analyses were performed using SPSS Statistics for Windows, Version 31.0.0.0 (IBM Corp., Armonk, New York, United States).

Ethics

This study protocol was approved by the Ethics Committee of Oita University Faculty of Medicine (Approval 3335). An opt-out approach was used, allowing patients to decline participation through the Department of Obstetrics and Gynecology, Oita University Faculty of Medicine website (https://og-oita.jp/family/). The study was conducted in accordance with the principles of the Declaration of Helsinki.

## Results

Table 1 summarizes the baseline, treatment, and outcome characteristics of the study population. The obstruction site was multiple or diffuse (not otherwise specified (NOS)) in approximately one-fourth of patients. The median age of the study population was 61 (interquartile range (IQR), 48-69). Approximately two-thirds of patients had received some form of anti-tumor therapy (antitumor agents, immune checkpoint inhibitors, antiangiogenic agents, radiation therapy, or their combination) for the primary disease within six weeks of MBO onset. Of the patients, 26.2% underwent surgical and/or endoscopic intervention (PEG in three, BS in three, SF in eight, MS in five), and 68.9% of patients resumed oral intake after MBO treatment (liquid in three, solid in 28). Although 35.6% of patients were discharged home with home-visiting care, the remaining patients were discharged to settings other than home (hospice in 21, community hospital in three, in-hospital death in five).

**Table 1:** Baseline, treatment, and outcome characteristics in the study population (N=45) * Ovary includes the fallopian tube and primary peritoneal cancer. † Not otherwise specified (multiple and/or diffuse). ‡ Chemotherapy includes maintenance therapy. CTCAE, Common Terminology Criteria for Adverse Events; MBO, malignant bowel obstruction

Variables	Frequency	Percentage
Age (years)		
< 65	29	64.4
≥ 65	16	35.6
Performance status		
< 3.0	20	44.4
≥ 3.0	25	55.6
Primary site		
Uterine cervix	15	33.3
Uterine corpus	13	28.9
Ovary *	17	37.8
Obstruction site		
Gastric outlet	2	4.4
Small bowel	26	57.8
Large bowel	5	11.1
NOS †	12	26.7
Chemotherapy within 6 weeks ‡		
No	19	42.2
Yes	26	57.8
Albumin level, g/dL		
< 3.0	23	51.1
≥ 3.0	22	48.9
Hemoglobin level, g/dL		
< 10.0	22	48.9
≥ 10.0	23	51.1
Ascites (CTCAE v5.0 criteria)		
< Grade 3	30	66.7
≥ Grade 3	15	33.3
Treatment type		
Medical	29	47.5
Surgical/endoscopic	16	26.2
Death within 90 days of MBO onset		
No	19	42.2
Yes	26	57.8
Resume oral intake		
No	14	31.1
Yes	31	68.9
Discharge home		
No	29	64.4
Yes	16	35.6

Variables	Frequency	Percentage
Age (years)		
< 65	29	64.4
≥ 65	16	35.6
Performance status		
< 3.0	20	44.4
≥ 3.0	25	55.6
Primary site		
Uterine cervix	15	33.3
Uterine corpus	13	28.9
Ovary *	17	37.8
Obstruction site		
Gastric outlet	2	4.4
Small bowel	26	57.8
Large bowel	5	11.1
NOS †	12	26.7
Chemotherapy within 6 weeks ‡		
No	19	42.2
Yes	26	57.8
Albumin level, g/dL		
< 3.0	23	51.1
≥ 3.0	22	48.9
Hemoglobin level, g/dL		
< 10.0	22	48.9
≥ 10.0	23	51.1
Ascites (CTCAE v5.0 criteria)		
< Grade 3	30	66.7
≥ Grade 3	15	33.3
Treatment type		
Medical	29	47.5
Surgical/endoscopic	16	26.2
Death within 90 days of MBO onset		
No	19	42.2
Yes	26	57.8
Resume oral intake		
No	14	31.1
Yes	31	68.9
Discharge home		
No	29	64.4
Yes	16	35.6

**Figure 2:**
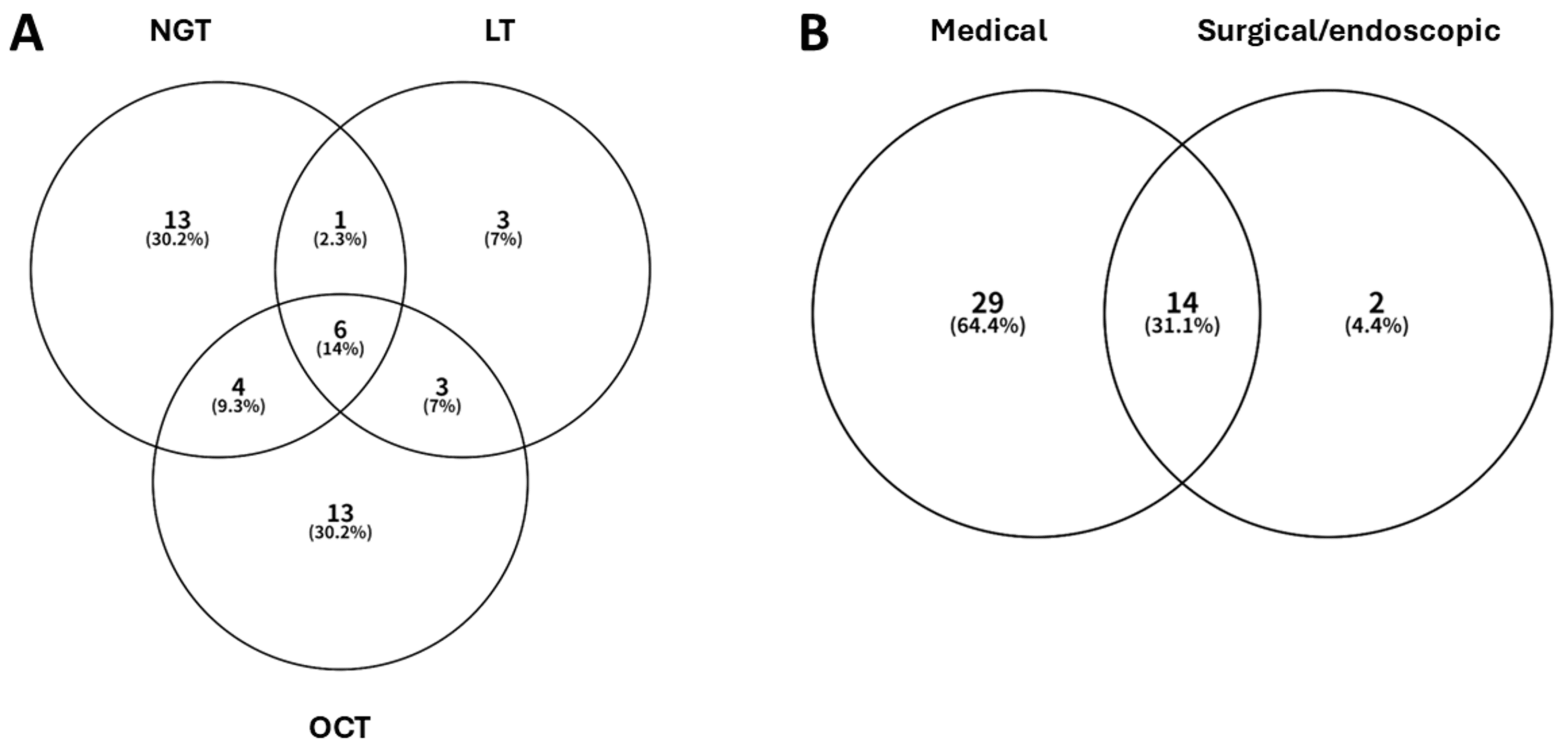
Venn diagrams illustrate the overlap between patients receiving medical treatment (A) and surgical/endoscopic treatment (B). NGT, nasogastric tube; LT, long intestinal tube; OCT, octreotide.

In clinical practice, medical and surgical/endoscopic treatments are often used concurrently. Therefore, we first examined the details of the treatment the patients underwent. Figure 2A demonstrates the details of medical treatment and its overlap for patients with MBO. Of the 45 patients, 95.9% (n=43) received some form of medical therapy. Octreotide (n = 26), NGT (n = 24), or their combination (n = 10) were the most frequent. Of the 43 patients, 14 (32.6%) received combination medical treatment, whereas 29 (67.4%) received a single medical treatment. Figure 2B summarizes the treatment overlap of medical and surgical/endoscopic treatment. Sixteen patients of the 45 (35.5%) received surgical or endoscopic treatment. Three patients received a combination surgical/endoscopic treatment: MS and PEG in two; BS and SF in one (data not shown). Fourteen of the 45 patients (31.1%) received concurrent medical and surgical/endoscopic treatment. No perioperative complications were observed.

**Figure 3:**
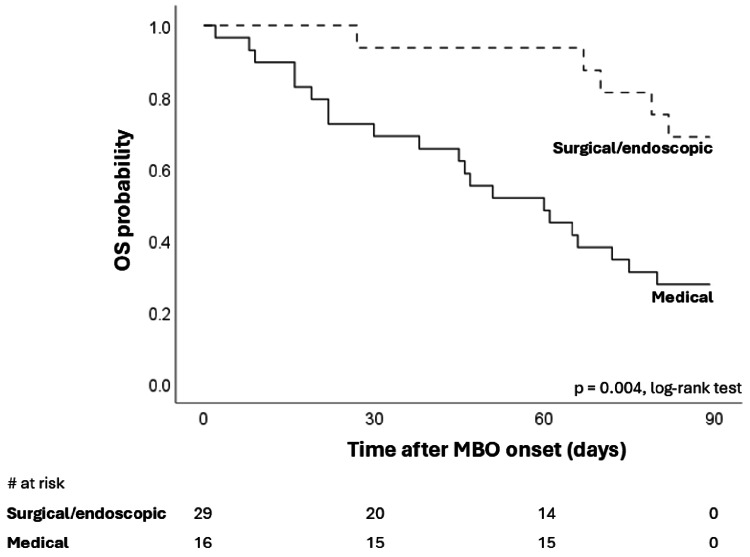
Overall survival stratified by treatment (surgical/endoscopic vs. medical). OS, overall survival; MBO, malignant bowel obstruction.

We then divided our cohort into two groups: patients who underwent medical treatment only (medical group) and those who received surgical/endoscopic treatment (surgical/endoscopic group), regardless of the combination of medical treatments. We conducted this analysis to assess whether surgical/endoscopic intervention adversely affects survival in patients with MBO. Because patients with MBO usually manifest as critically ill, such as carcinomatosis and cachexia. Figure 3 shows a significant difference in 90-day survival by treatment type. Median overall OS was 60 days (95%CI: 35-85 days) in the medical treatment group and 116 days (95%CI: 100-132 days) in the surgical/endoscopic treatment group. Contrary to our expectations, surgical/endoscopic treatment was associated with improved survival compared with medical treatment alone.

Next, we assessed the treatment outcome of MBO by treatment type. The results are summarized in Table 2. The proportion of patients discharged home after oral intake is significantly higher in the surgical/endoscopic group. All patients who underwent any surgical/endoscopic treatment resumed oral intake.

**Table 2:** Outcomes by treatment in the study population (N=45)

Variables	Outcomes	Medical (n = 29), n (%)	Surgical/ endoscopic (n = 16), n (%)	p-value	Chi-square value
Resume oral intake	No	14 (100)	0 (0)	< 0.001	11.212
	Yes	15 (48.4)	16 (51.6)		
Discharge home	No	24 (82.8)	5 (17.2)	< 0.001	11.939
	Yes	5 (31.3)	11 (68.8)		

**Figure 4:**
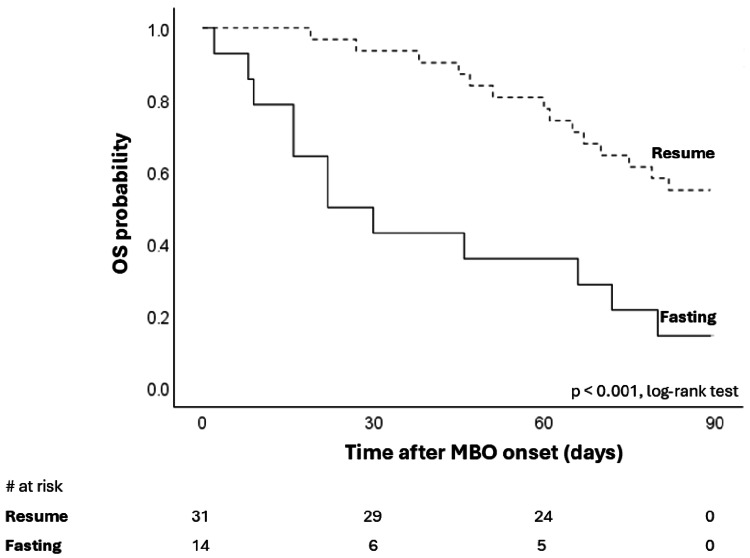
Overall survival stratified by oral intake after treatment for MBO (resumed vs. fasting). OS, overall survival; MBO, malignant bowel obstruction.

**Figure 5:**
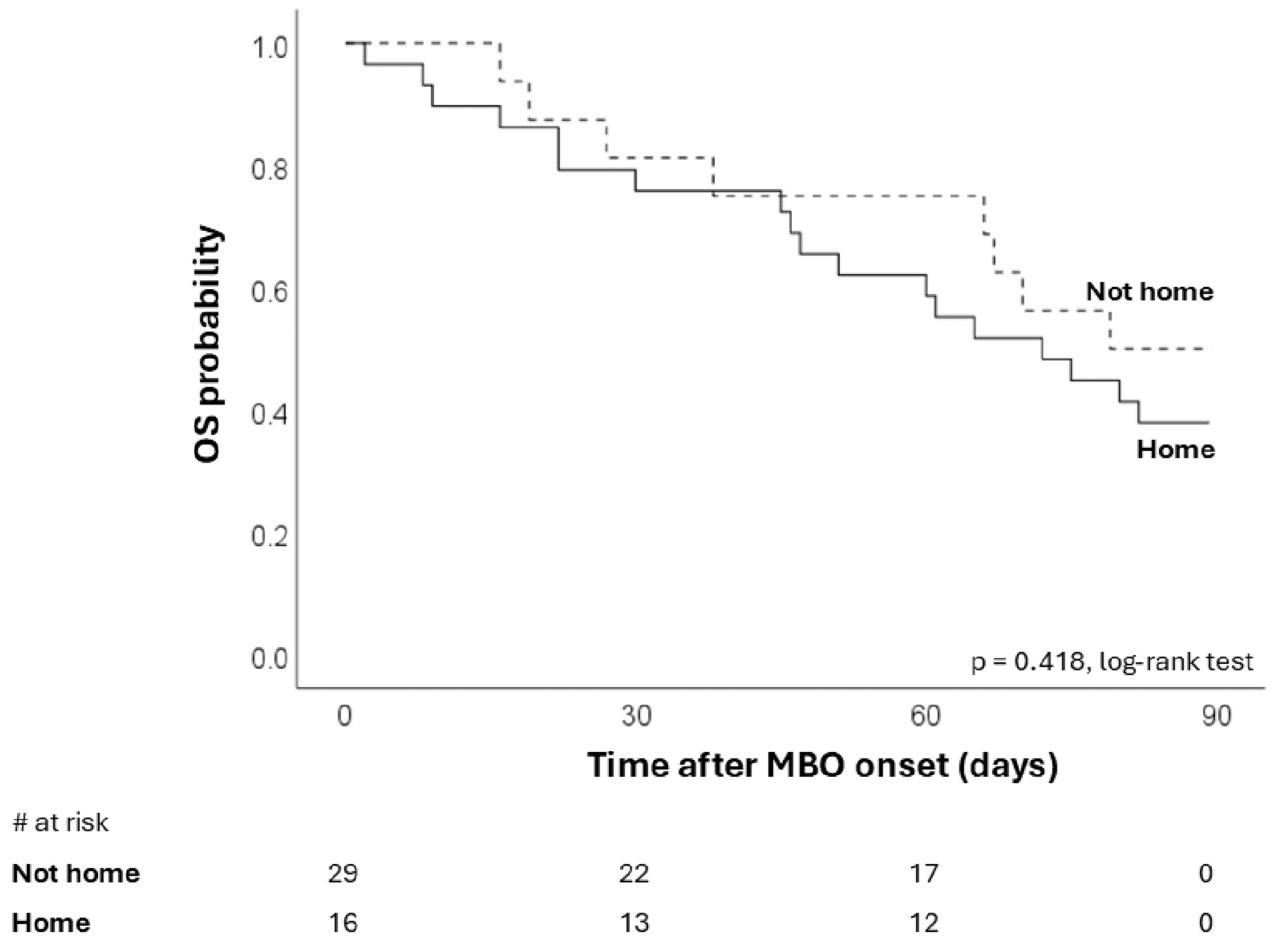
Overall survival stratified by the place of discharge (not home vs. home). OS, overall survival; MBO, malignant bowel obstruction.

Furthermore, we assessed whether the aforementioned differences affected patients' survival time with MBO. Figure 4 shows a significant difference in 90-day survival associated with oral intake outcome. Median overall OS was 22 days (95%CI: 5-39 days) in the fasting group and 105 days (95%CI: 65-145) in the resumed oral intake group. Figure 5 shows no significant difference in OS by the destination of discharge. Median OS was 72 days (95%CI: 47-97 days) in the not-home group and 79 days (95%CI: not acceptable) in the home group. Discharge location was not associated with survival in patients with MBO.

Additionally, we performed univariate and multivariable logistic regression analyses, as well as a Cox proportional hazards model. Before this analysis, we recategorized the primary site and obstruction site (both are initially shown in Table 1) into dichotomous variables. The primary site was recategorized as the uterus (n = 28) or the ovary (n = 17). The obstruction site was categorized as NOS (n = 12) or gastric/small/large (n = 33). Then, we used nine variables to assess their impact on 90-day survival after MBO onset (age, PS, primary site, obstruction site, therapy for the disease within six weeks, albumin, hemoglobin, ascites, and treatment type). To select variables, we employed forward selection based on the likelihood ratio test, given the limited number of subjects in this study. Table 3 presents the results of the Cox proportional hazards model for factors associated with 90-day survival by univariate analysis. Obstruction site of NOS (multiple or diffuse), albumin level ≥ 3.0, and surgical/endoscopic treatment were associated with improved survival compared with each reference category. Hence, the presence of ascites ≥ Grade 3 was associated with poorer survival than < Grade 3.

**Table 3:** Factors associated with 90-day survival in patients with MBO by univariate analysis. * The hazard ratio is the ratio of hazard for each category to the reference category. † Ovary includes the fallopian tube and primary peritoneal cancer. ‡ Not otherwise specified (multiple and/or diffuse). § Chemotherapy includes maintenance therapy. CTCAE, Common Terminology Criteria for Adverse Events; MBO, malignant bowel obstruction

Variables	Hazard Ratio*	95% CI	P-value	Chi-square value
Age, years				
< 65	1.00			
≥ 65	1.417	0.650–3.093	0.381	0.775
Performance status				
< 3.0	1.00			
≥ 3.0	1.253	0.575–2.731	0.570	0.324
Primary site				
Uterus	1.00			
Ovary ^†^	0.876	0.483–2.351	0.876	0.025
Obstruction site				
Gastric/small/large	1.00			
NOS ^‡^	0.362	0.163–0.804	0.013	6.750
Chemotherapy within 6 weeks ^§^				
No	1.00			
Yes	1.024	0.470–2.231	0.953	0.003
Albumin level, g/dL				
< 3.0	1.00			
≥ 3.0	0.445	0.201–0.986	0.046	4.195
Hemoglobin level, g/dL				
< 10.0	1.00			
≥ 10.0	0.574	0.263–1.253	0.164	1.990
Ascites (CTCAE v5.0 criteria)				
< Grade 3	1.00			
≥ Grade 3	2.563	1.170–5.617	0.019	5.921
Treatment type				
Medical	1.00			
Surgical/endoscopic	0.257	0.096–0.687	0.007	8.461

Table 4 presents the results of the Cox proportional hazards model for factors associated with 90-day survival by multivariate analysis. Surgical/endoscopic treatment was associated with improved survival compared with medical therapy alone. In contrast, the presence of ascites ≥ Grade 3 was associated with poorer survival than < Grade 3. 

**Table 4:** Factors associated with 90-day survival in patients with MBO by multivariate analysis. * The hazard ratio is the ratio of hazard for each category to the reference category. CTCAE, Common Terminology Criteria for Adverse Events; CI, Confidence interval. Statistical test used: Chi-square test: chi-square value 12.769, significance level: p < 0.05.

Variables	Hazard Ratio*	95% CI	p-value
Treatment			
Medical	1.00	0.104–0.754	0.012
Surgical/endoscopic	0.280		
Ascites (CTCAE v5.0 criteria)			
< Grade 3	1.00	1.015–4.998	0.046
≥ Grade 3	2.252		

**Figure 6:**
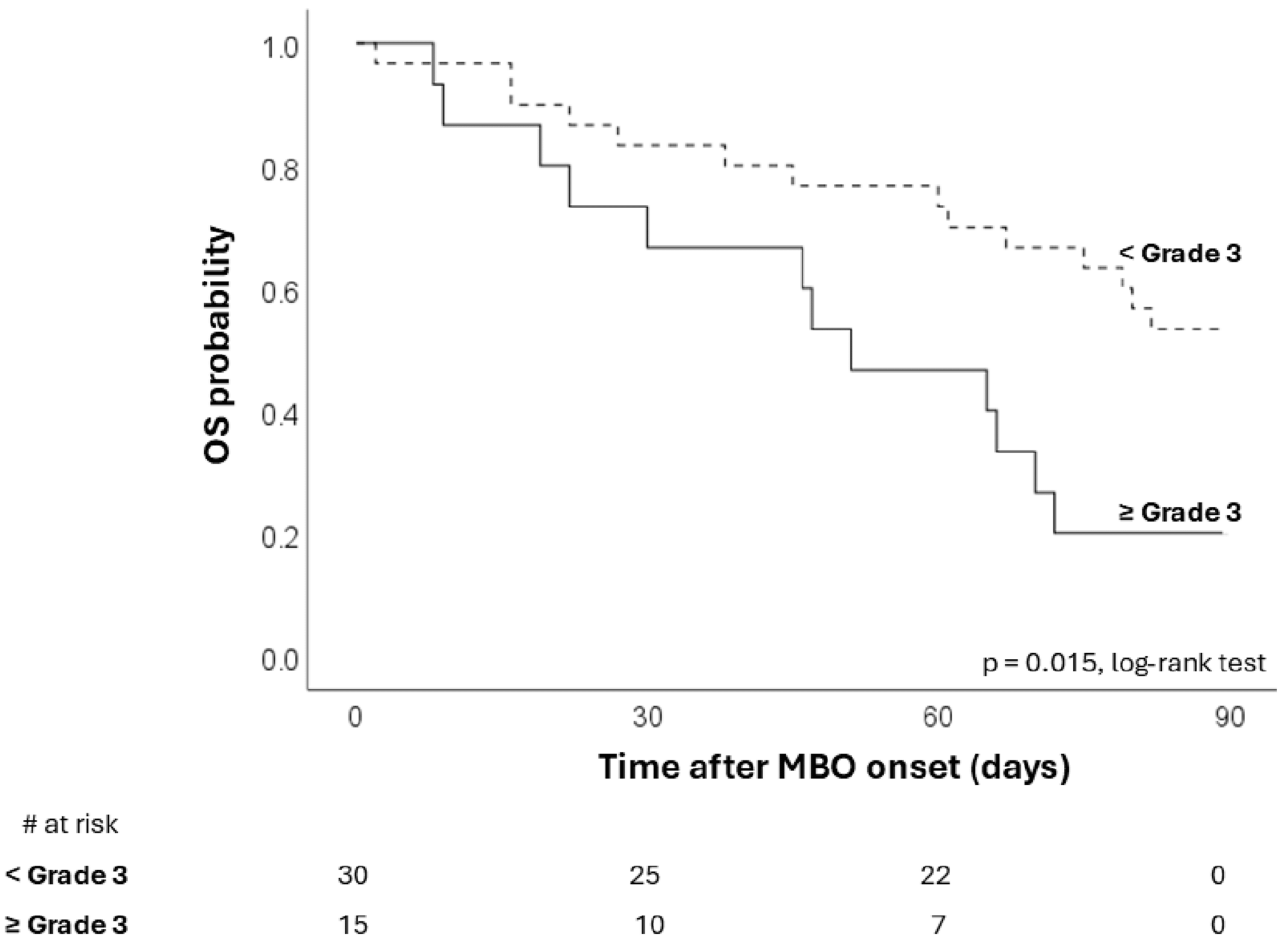
Overall survival stratified by ascites severity (< Grade 3 vs. ≥ Grade 3). OS, overall survival; MBO, malignant bowel obstruction.

Figure 6 shows a significant difference in 90-day survival stratified by ascites severity. Median overall OS was 51 days (95%CI: 27-75 days) in the patients with ascites ≥ Grade 3 and 91 days (95%CI: 45-37 days) in the patients with ascites < Grade 3. The severity of ascites was associated with survival in patients with MBO.

## Discussion

Although MBO management can be among the most challenging aspects of late-stage gynecologic cancer care, its details have remained unclear because of its highly individualized nature. In the present study, we aimed to assess treatment for MBO, particularly the impact of surgical/endoscopic interventions on patient survival. Our study yielded two critical clinical findings. First, in multivariate analysis, surgical/endoscopic interventions are associated with more prolonged survival in MBO patients, whereas ascites severity is associated with shorter survival. Second, the proportion of patients who regain oral intake and are discharged home is higher among patients undergoing these interventions.

Our findings suggest that surgical/endoscopic treatment for patients with gynecological MBO does not worsen prognosis. These results are consistent with previous studies showing a better prognosis in patients undergoing surgical treatment for gynecological MBO than in those receiving conservative treatment [23,24]. However, reports indicate that patients who undergo colostomy have a high rate of death within 60 days [13]. Surgical treatment is certainly invasive, and treatment selection must be individualized to avoid worsening the prognosis [24]. Several factors have been proposed as contributing to improved prognosis with surgical treatment [7]. Our study found that ascites severity is a prognostic factor for poorer outcomes. In summary, these findings indicate that surgical and endoscopic treatment for MBO, when appropriate treatment is selected for each patient, can improve prognosis and quality of life at the end of life.

Our results are generally in line with other reports examining patients with gynecologic cancer undergoing surgery for MBO, where surgical intervention provides prolonged survival [24]. Some mechanisms underlying prolonged survival in the surgical/endoscopic treatment group should be considered. First, a clinician selects a surgical/endoscopic candidate after carefully weighing the risks and benefits to avoid perioperative complications that could be lethal. This means that patient selection bias is likely [25]. Second, 87.5% (14 of 16) of patients in the surgical/endoscopic group received medical treatment before or after surgery or endoscopy. Thus, medical treatment is not a substitute for surgical treatment but rather a component of the therapeutic strategy for MBO [4]. This is partially why we still have somewhat weak evidence supporting surgical management to prolong survival, as shown in a meta-analysis [26]. Finally, surgical/endoscopic interventions for patients with MBO are often conducted semi-electively. Although quantitative assessment was not feasible, rapid surgical decision-making through close collaboration among multidisciplinary teams could have contributed to our results [27].

In this study, we examined the epidemiology, management, and factors influencing survival in patients with gynecologic MBO who underwent medical and surgical/endoscopic treatment using data from 1,085 patients over 12 years. We found that the uterine cervix accounted for 33.3% of primary sites associated with MBO, the first time this has been reported. Goto et al. reported that, in a study of 53 Japanese patients who underwent surgical intervention for MBO alone, 17.0% of primary sites were the uterine cervix [28]. These findings suggest that the uterine cervix is a major primary site in Japan, whereas in Western European countries, it is not.

We found that surgical/endoscopic interventions for gynecologic MBO are associated with improved survival and a higher rate of discharge home. As gynecologic oncologists, we do not typically manage gastrointestinal organs. Therefore, we should usually consult gastrointestinal surgeons and gastroenterologists for the management of gynecologic MBO. We should also coordinate discharge planning with other healthcare professionals, including social workers and nurses. . Otherwise, we could miss the window of opportunity for surgical intervention and discharge home, as patients with late-stage cancer may be at risk of collapse. In this study, we examined the relationship between patient-reported well-being and health outcomes in a population that has not previously been studied. Although the samples drawn from the real-world clinical setting were diverse, the use of these brief patient-reported measures could be practical in busy primary care practices.

This study has four limitations that should be acknowledged. First, 95.5% of patients in the surgical/endoscopic group also received medical treatment before or concurrently with surgery or endoscopy. Selection bias toward surgical/endoscopic intervention might exist. Therefore, the results obtained in this study assess solely the efficacy and safety of medical and surgical/endoscopic interventions. Second, the 12-year reporting period does not reflect recent developments in treatment options for patients with MBO. Primarily, the on-label MS for patients with MBO has been available in Japan since 2012, but it has taken some time to be widely adopted in daily practice [29]. Additionally, OCT was the only agent incorporated into the Japanese healthcare system for the treatment of MBO [30]. However, other drugs, such as haloperidol, metoclopramide, olanzapine, and scopolamine, are often used to relieve nausea and vomiting in patients with MBO. The use and effects of these drugs were not included in this study. Third, we designed the proportions of recovery of oral intake and of discharge home as secondary outcome measures in this study. Some studies report that these two factors are essential to the quality of end-of-life care for patients with cancer [19,28]. However, subjective-level parameters, such as patient-reported outcomes, are lacking due to the retrospective nature of this study. Finally, the small sample size available for final analysis reduced the statistical robustness of the Cox regression model, resulting in a small number of variables in the regression model and an incalculable 95% CI for some assessments.

## Conclusions

In this retrospective analysis of gynecologic patients with MBO, multivariable analysis found that surgical/endoscopic interventions were associated with more prolonged survival after MBO onset. In contrast, ascites severity is associated with shorter survival. Additionally, surgical/endoscopic interventions are associated with a higher proportion of patients who resume oral intake and are discharged home compared with medical treatment alone. Although a larger, well-designed prospective study is required, these findings should help physicians who face the challenge of shared decision-making with patients with MBO at the end of life.
